# Characteristics of the menstrual cycle and hormonal contraceptive use in elite Spanish basketball players

**DOI:** 10.3389/fspor.2025.1642415

**Published:** 2025-10-29

**Authors:** Pilar Crespo, Nuria Herranz, Víctor Estella, Esther Morencos, María Herranz, Gil Rodas

**Affiliations:** ^1^ Helse Fisioterapia, Zaragoza, Spain; ^2^ Medical Services, Spanish Basketball Federation, Madrid, Spain; ^3^ Exercise Physiology Group, Exercise and Sport Sciences, Faculty of Health Sciences, Universidad Francisco de Vitoria, Madrid, Spain; ^4^ Preventive Medicine Service, Hospital Alvaro Cunqueiro, Vigo, Spain; ^5^ Sports and Exercise Medicine Unit, Hospital Clinic y San Joan de Dèu, Barcelona, Spain; ^6^ FC Barcelona Medical Department (FIFA Medical Excellence Center), Barcelona, Spain

**Keywords:** women’s basketball, menstrual cycle, premenstrual syndrome, menstrual symptoms, hormonal contraceptives

## Abstract

**Introduction:**

Previous research has shown how menstrual-related symptoms (e.g., abdominal cramps, tiredness/fatigue) can limit athletes’ ability to train. Nonetheless, the specific characteristics of the menstrual cycle (MC) and their relationship with performance, well-being, and injuries in athletes are not well understood. This cross-sectional study aimed to better understand the characteristics of the MC (menarche, length, dysmenorrhea, amenorrhea, etc.) and the use of hormonal contraceptives among the elite Spanish basketball players.

**Methods:**

A total of 228 healthy elite female basketball players from both first division and second division of the Spanish league were included in this cross-sectional study. Subjects were assessed for MC characteristics using a validated questionnaire.

**Results:**

The menarche age in the study population was 13.21 ± 1.55 years. The majority of players (78.51%) experienced a regular MC of 27.69 ± 3.78 days, with a range from 17 to 40 days. One hundred ninety-eight out of the 228 players surveyed (86.84%) experienced dysmenorrhea during their periods, and 41.67% took pain medication. Moreover, 77.63% of the players showed symptoms of premenstrual syndrome. Amenorrhea was also reported by 22.81% of them, and 3.51% of the players had been pregnant at some point in their lives. No statistically significant differences were observed when comparing the characteristics of the MC between first and second division players. Less than a quarter of the studied population referred using hormonal contraceptives regularly.

**Conclusion:**

MC alterations are prevalent among female basketball players. More research is necessary to understand how this can affect the quality of life, the performance, and the injury susceptibility of female athletes.

## 1 Introduction

Athlete performance in both individual and team sports is influenced by various factors, extensively analyzed in the literature. However, most of these publications focus on male athletes, often assuming the findings apply equally to women (1). The growing professionalism and visibility of women's sports, such as soccer in Spain, has led to an increase in scientific publications focusing on female athletes (2). Recent studies have begun to investigate the prevalence of injuries in women's sports, and the possible gender-specific causes, such as anatomical, biomechanical, and hormonal factors (3, 4).

One of the most significant physiological differences between women and men is the menstrual cycle (MC), which entails hormonal fluctuations that can influence performance (5–8). The onset of these hormonal fluctuations is marked by menarche, the first menstruation. On average, in Norway, menarche occurs around the age of 13 in non-athletic adolescents and slightly later in athletes (around 13.4 years) (9). It is well documented that the MC can significantly impact the daily life of women, both physically and psychologically, often affecting their quality of life (10). More than 70% of women experience symptoms during the premenstrual phase, known as premenstrual syndrome (PMS) (10, 11). These symptoms include mood swings, fatigue, and physical discomfort (10). Additionally, between 16% and 91% of women in their reproductive age experience primary dysmenorrhea, a chronic, cyclical pain occurring before and/or during menstruation without any underlying pathology (12). Severe disabling pain is reported by 2–29% of these women (12).

Although the primary function of hormonal contraceptives (HCs) is contraception, they are also used to manage the symptoms of PMS, dysmenorrhea, and other conditions, and are also used to skip periods at times when the symptoms associated with MC can be very limiting, like during important games; without being exempt from non-negligible side effects (13). In the general population, about 30% of women use HCs (14). This percentage is notably higher in athletes. Studies have shown that nearly 70% of female athletes have used HCs at some point, with around 50% using them at the time of the survey (15, 16). This higher rate of use may indicate that menstrual symptoms are more common or severe in athletes.

Although scientific literature addressing MC in athletes is emerging, the existing studies present several limitations. The results have been inconsistent largely due to very small populations (“n” around 15) or the inclusion of women with varying and often undefined levels of physical activity, ranging from occasional recreational exercise to elite athletic performance (17). Additionally, most of these studies do not explore the characteristics of the MC in detail, nor do they comprehensively examine the concurrent use of HCs. A better understanding of the physical effects of the MC on female athletes could lead to more tailored training strategies, helping to minimize any potential negative impact of the MC on performance.

Therefore, we considered it necessary to carry out a more extensive study, focusing on an elite sports population in Spain. Since basketball is the sport with the largest number of women's sport licenses in the country (18), we decided to conduct the research among players of the two main basketball leagues in our country: the first women's League (LF1): *Liga Femenina Endesa*, and the second women's League (LF2), now known as the *Liga Femenina Challenge*. Some of the LF1 teams also participate in European competitions (LF1E).

The main objective of the study is to determine the characteristics of the MC among the LF1 and LF2 players. As secondary objectives we propose to identify the presence of gynecological alterations among the study group, to calculate the pregnancies rate in the study group and to review the use of HCs within those Spanish basketball leagues.

## 2 Material and methods

### 2.1 Study design

This study is a cross-sectional, descriptive study of the MC characteristics among female basketball players from the national Spanish basketball leagues during the 2019–2020 season.

### 2.2 Study population

Elite basketball players from 42 teams from LF1 and LF2 were invited to participate in this study.

**Figure 1:**
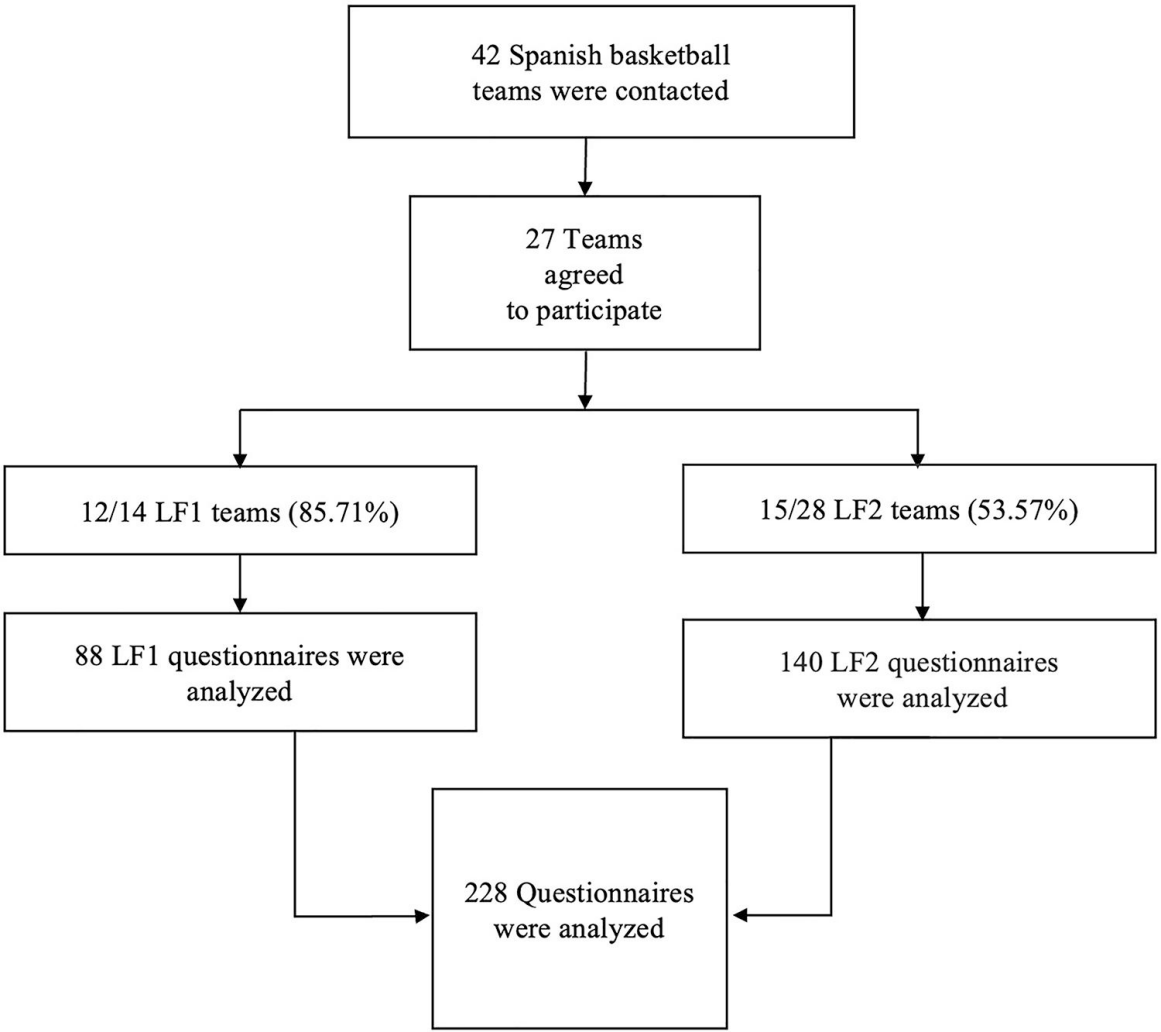
Population description flow chart. LF1: Spain's first national women's basketball league. LF2: Spain's second national women's basketball league.

Inclusion criteria were participation in one of the 42 teams during the 2019/20 season, being 18 years of age or older at the end of data collection, providing informed consent, and completing the questionnaire correctly. Players who failed to meet any inclusion criteria were excluded (Figure 1).

### 2.3 Study design and data collection

The medical services of the 42 clubs from both first- and second-division leagues were contacted to explain the study's purpose and logistics.

A specific questionnaire was developed to assess the MC characteristics among basketball players. The questionnaire was based on available scientific evidence and validated by a panel of gynecologists and sports physicians.

Originally developed in Spanish, the questionnaire was translated into English and French. It was tested by basketball players from foreign leagues (see supplementary material). The reproducibility of the questionnaire was evaluated achieving a congruence of 92.11%.

The questionnaire consisted of 24 items that allowed the collection of demographic information (league, age, height, race, handedness, playing position, and regular medication use), MC data (age at menarche, regularity and length of the MC, heavy flow, dysmenorrhea and other symptoms during the MC, use of pain medication during menstruation, premenstrual symptoms, amenorrhea and its cause, pregnancy history, gynecological or endocrine disorders, and gynecological habits), and data on the use of HCs. Some questions allowed more than one answer.

The informed consent form and a link to the questionnaire were sent to the participating teams’ medical services. The medical services were responsible for distributing them to the players. Participants completed the coded questionnaire online, and the collected data were transcribed into an Excel sheet for analysis.

All the participants provided written informed consent, which was collected through the medical services of each club before participation.

### 2.4 Statistical analysis

The statistical analysis was conducted using the open source packages based on the programming language Python (version 3.9.16). The packages used were Pandas to manage the tabular data, NumPy for vectorial calculations, and SciPy (stats package) for statistical analyses.

Descriptive statistics were used to summarize quantitative variables (age, heigth, etc.), including measures of central tendency (mean) and dispersion (95% confidence interval). Qualitative variables (presence of dysmenorrhea, use of HCs, etc.), were summarized using frequency tables and proportions.

To stablish comparisons between players from LF1 and LF2, bivariate analysis was conducted using T-tests to compare means of continuous variables, and Chi-square (*χ*2) tests to compare proportions of qualitative variables.

In those cases where normality assumptions were not met, non-parametric tests were applied.

A 95% confidence level was considered in all analysis, and statistical significance was defined as a *p*-value less than 0.05.

## 3 Results

### 3.1 Description of the population

As shown in Figure 1, 27 out of 42 teams contacted (64.29%) agreed to participate in the study, including 12 teams from the LF1 (85.71% of LF1 teams) and 15 teams from the LF2 (53.57% of LF2 teams). Among the 12 LF1 teams, 3 teams (25.00%) also played in European competitions (LF1E) during the study season, while the other 9 teams (75.00%) only competed in the national league.

A total of 249 questionnaires answers out of 289 possible were received (response rate: 86.10%). However, 21 questionnaires (8.43%) were excluded due to repetition (10 questionnaires), improper completion (1 questionnaire), or failure to meet the inclusion criteria (10 questionnaires). Data from 228 players were included in the final analysis: 88 players (38.60%) from LF1 and 140 players (61.40%) from LF2 (Figure 1). Of the LF1 players, 34 players (38.64%) were part of the LF1E subgroup, while 54 players (61.36%) played only in the national league.

The demographic characteristics of the study population are summarized in Table 1. The average age of the players was 25.32 ± 4.78 years, with ages ranging from 18 to 45 years. The mean age of the LF2 players (24.49 ± 4.77 years) was significantly lower than the mean age of the LF1 players (26.67 ± 4.54 years) (*p* = 0.0005).

**Table 1:** General description of the population.

		Total		LF1		LF2		*P* (LF1/LF2)
		*n* (228)		*n* (88)		*n* (140)		
Age (years)		25.32 ± 4.78 range: 18–45		26.67 ± 4.54 range: 18–40		24.49 ± 4.77 range: 18–45		0.0005
Height (cm)	Global	179.83 ± 8.02 range: 159–196		181.64 ± 7.78 range: 166–196		178.69 ± 7.98 range: 159–196		0.0061
	Point guard	170.50 ± 5.18 range: 159–183		172.00 ± 4.47 range: 166–182		169.58 ± 5.44 range: 159–183		0.1255
	Shooting guard	175.89 ± 4.52 range: 165–188		177.58 ± 4.92 range: 168–188		174.85 ± 3.97 range: 165–184		0.0263
	Small forward	179.30 ± 4.3 range: 167–188		181.93 ± 3.67 range: 174–188		177.65 ± 3.96 range: 167–183		0.0018
	Power forward	185.80 ± 4.2 range: 176–196		188.00 ± 3.71 range: 178–196		184.10 ± 3.81 range: 176–192		0.0004
	Center	190.74 ± 3.49 range: 184–196		191.80 ± 2.90 range: 18 −196		189.62 ± 5.46 range: 184–196		0.2141
		*n* (228)	%	*n* (88)	%	*n* (140)	%	*P* (LF1/LF2)
Race	Caucasian	190	83.33	68	77.27	122	87.14	0.067
	Asian	1	0.44	0	0	1	0.71	0.38
	Afro American	36	15.79	19	21.59	17	12.14	0.063
	South American	1	0.44	1	1.14	0	0	1.0
Handedness	Right-handed	203	89.04	77	87.50	126	90.00	0.66
	Left-handed	13	5.70	5	5.68	8	5.71	1.0
	Ambidextrous	11	4.82	6	6.82	5	3.57	0.34
	N/A	1	0.44	0	0	1	0.72	NA
Position	Point guard	42	18.42	16	18.18	26	18.57	1.0
	Shooting guard	63	27.63	24	27.27	39	27.86	1.0
	Small forward	38	16.67	15	17.05	23	16.43	1.0
	Power forward	54	23.68	23	26.14	31	22.14	0.52
	Center	31	13.60	10	11.36	21	15.00	0.55
Chronic medication unrelated to menstrual cycle	Yes	25	10.96	9	10.23	16	11.43	0.83
	No	203	89.04	79	89.77	124	88.57	0.83

LF1: Spain's first national women's basketball league. LF2: Spain's second national women's basketball league. N/A, no answer.

The height of the players ranged from 159 to 196 cm, with a mean height of 179.83 ± 8.02 cm. As shown in Table 1, the height varied depending on the position, with taller players found in the center position.

LF1 players were on average 3 cm taller than LF2 players (*p* = 0.0061), with statistically significant height differences found between small forwards (*p* = 0.0018), shooting guards (*p* = 0.0263) and power forwards (*p* = 0.0004) in LF1 and LF2.

Other characteristics such as race, handedness, position distribution, and use of chronic medication, were homogeneous between LF1 and LF2 groups, with no statistically significant differences (Table 1).

### 3.2 Menstrual cycle characteristics

The average age of menarche among the players was 13.21 ± 1.55 years, as shown in Table 2. Significant differences were observed between players’ positions, with point guards reporting a younger menarche age (12.86 ± 1.49 years) compared to centers (13.73 ± 1.78 years) (*p* = 0.02).

**Table 2:** Menstrual cycle characteristics.

		Total		LF1		LF2		*P* (LF1/LF2)
Mean age of menarche (years)	Global	13.21 ± 1.55 range: 9–18		13.26 ± 1.48 range: 10–17		13.18 ± 1.60 range: 9–18		0.70
	Point guard	12.86 ± 1.49 range: 10–17		12.75 ± 1.34 range: 11–16		12.92 ± 1.60 range: 10–17		0.70
	Shooting guard	13.17 ± 1.57 range: 10–17		13.74 ± 1.63 range: 10–17		12.80 ± 1.43 range: 10–15		0.078
	Small forward	13.24 ± 1.78 range: 10–18		12.73 ± 1.58 range: 10–15		13.57 ± 1.85 range: 11–18		0.14
	Power forward	13.26 ± 4.49 range: 9–16		12.57 ± 1.20 range: 12–16		13.03 ± 1.38 range: 9–16		0.13
	Center	13.73 ± 1.78 range: 11–17		12.83 ± 1.83 range: 11–16		14.00 ± 1.72 range: 11–17		0.79
Mean menstrual cycle duration (days)		27.69 ± 3.78 (range: 17–40)		27.62 ± 4.63 (range: 17–40)		27.73 ± 3.16 (range: 21–40)		0.83
		*n* (228)	%	*n* (88)	%	*n* (140)	%	*P* (LF1/LF2)
Regular cycle	Yes	179	78.51	71	80.68	108	77.14	0.62
	No	49	21.49	17	19.32	32	22.86	
Normal length period (3–7 days)	Yes	180	78.95	72	81.82	108	77.14	0.5
	Sometimes	24	10.53	4	4.54	20	14.29	0.025
	No	24	10.53	12	13.64	12	8.57	0.26
Heavy menstrual bleeding	Yes	47	20.61	19	21.59	28	20.00	0.86
	Sometimes	80	35.09	31	35.23	49	35.00	1.0
	No	101	44.30	38	43.18	63	45.00	0.89
Dysmenorrhea	Yes	99	43.42	38	43.18	61	43.57	1.0
	Sometimes	99	43.42	37	42.05	62	44.29	0.78
	No	29	12.72	13	14.77	16	11.43	0.54
	N/A	1	0.44	0	0	1	0.71	1.0
Other symptoms during period	Yes	173	75.88	64	72.73	109	77.86	0.42
	No	55	24.12	24	27.27	31	22.14	
Medication during period	Yes	95	41.67	33	37.50	62	44.29	0.33
	No	133	58.33	55	62.50	78	55.71	
PMS	Yes	177	77.63	68	77.27	109	77.86	1.0
	No	51	22.37	20	22.73	31	22.14	
Amenorrhea (skipped 2 or more periods)	Sometimes	52	22.81	23	26.14	29	20.71	0.41
	Never	175	76.75	65	73.86	110	78.57	0.42
	N/A	1	0.44	0	0	1	0.71	1.0
Pregnancies	No	220	96.49	84	95.45	136	97.14	0.48
	1	4	1.75	2	2.27	2	1.43	0.64
	2	3	1.32	1	1.14	2	1.43	1.0
	3	1	0.44	1	1.14	0	0	0.38
Gynecological disorders	Yes	10	4.39	5	5.68	5	3.57	0.54
	No	218	95.61	83	94.32	135	96.43	
Gynecological checkups	Annual	83	36.40	38	43.18	45	32.14	0.11
	Every two years	29	12.72	8	9.09	21	15.00	0.22
	When I have a problem	68	29.83	29	32.95	39	27.86	0.45
	Never	47	20.61	12	13.64	35	25.00	0.04
	N/A	1	0.44	1	1.14	0	0	0.38

LF1: Spain's first national women's basketball league. LF2: Spain's second national women's basketball league. N/A, no answer; PMS, premenstrual syndrome.

Most players (78.51%) reported regular MCs with an average of 27.69 ± 3.78 days, ranging from 17 to 40 days. In contrast to that, 21.49% of players experienced irregular cycles, ranging from 15 to 120 days (Table 2). Players in the LF1E group were the most regular, with 88.24% reporting regular cycles.

A total of 21.06% of the players experienced periods lasting more than 7 days, while 78.95% had normal-length periods (3–7 days). Heavy menstrual flow was common, with 20.61% of players reporting it regularly and 35.09% occasionally (Table 2).

Dysmenorrhea affected 86.84% of the players, with half of them experiencing it only in some periods, and the other half during every period (Table 2). Among the players without dysmenorrhea (12.72%), 2.19% reported that they had previously experienced the condition but had not felt pain for at least six cycles.

Most players who reported dysmenorrhea felt pain on the first day of their period (34.21% of the study population), while 44.30% experienced it during both the first and second days. Only 3.95% of the players experienced dysmenorrhea during their entire period.

**Figure 2:**
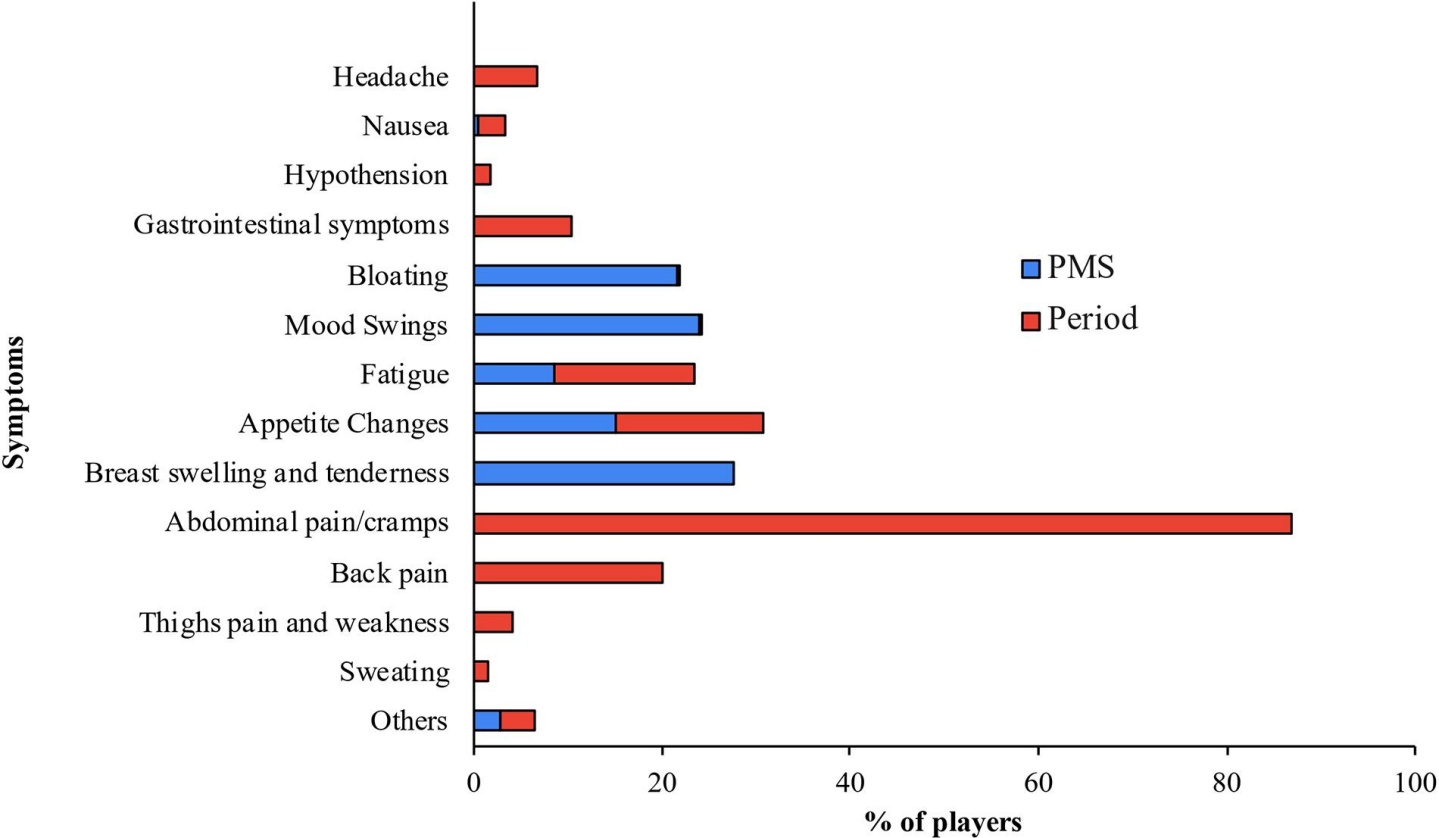
Plot showing the prevalence of symptoms during menstruation (period) and premenstrual syndrom (PMS).

Additionally, 75.88% of the players reported other symptoms during menstruation (Table 2), with back pain and appetite changes being the most common symptoms described (Figure 2). Nearly half of the players (41.67%) referred taking pain medication during menstruation (Table 2), being non-steroidal anti-inflammatory drugs the most common choice (93.68%).

Premenstrual syndrome symptoms were reported by 77.63% of the players (Table 2), with breast swelling and tenderness (27.49%), and mood swings (23.99%) being the most frequently mentioned. Considering menstrual and premenstrual symptoms together, the most repeated symptom after dysmenorrhea was appetite changes (Figure 2).

As is shown in Table 2, the occurrence of amenorrhea (missing two or more consecutive periods) at some point in the menstrual life was reported by 52 players (22.81%), with nearly half of the players (42.59%) unable to identify the cause. Physical or mental stress was cited as the primary reason of amenorrhea by 29.63% of players, particularly among the LF1E players, were 71,43% of them marked mental stress as the primary reason of amenorrhea. Other causes of amenorrhea included sudden weight loss (11.11%).

Eight players (3.51%) had been pregnant (Table 2).

Ten players (4.39%) had a gynecological disorder (Table 2), including polycystic ovary syndrome (6 players), ovary cysts (2 players), and bartholinitis (one case).

Less than half (49.12%) of the players had regular gynecological checkups (annual or every two years), while 29.83% only visited the gynecologist when they had a problem. Moreover, 25.00% of the LF2 players had never had a gynecological checkup. As is shown in Table 2, this percentage decreases to 13.64% in the LF1 group, a difference that is statistically significant (*p* = 0.04).

No other relevant statistically significant differences were found in MC characteristics between LF1 and LF2 players.

### 3.3 Hormonal contraceptive use

Details of HCs use are presented in Table 3.

**Table 3:** Use of hormonal contraceptives.

	TOTAL		LF1		LF2		*P* (LF1/LF2)
	*n* (228)	%	*n* (88)	%	*n* (140)	%	
Yes	53	23.25	24	27.27	29	20.71	0.26
No	175	76.75	64	72.73	111	79.29	
Oral	40	74.07	17	70.83	23	76.67	0.75
Non-oral	14	25.93	7	29.17	7	23.33	

LF1: Spain's first national women's basketball league. LF2: Spain's second national women's basketball league. N/A, no answer. The first two lines show the use of hormonal contraceptives (HCs) among the studied players. The last two lines indicate the preferred HCs among those players who answered yes on the previous question.

Fifty-three out of 228 players (23.25%) used HCs regularly. The most used method was the oral pill, being the preferred choice for 74.07% of the players. The combined pill was the selected option for 85.00% of these players. Among players using non-oral contraceptive methods, the contraceptive ring was the most popular option (64.29%).

No statistically significant differences in HCs use were observed between LF1 and LF2 players.

## 4 Discussion

This study aimed to evaluate the MC characteristics and HCs use among elite female basketball players in Spain.

As shown in the results, the players age of menarche was slightly delayed, and a higher prevalence of dysmenorrhea and heavy menstrual bleeding was observed compared to the general population. A quarter of the players used HCs, with the oral pill being the most common contraceptive. The MC characteristics and HCs usage were similar between LF1 and LF2 players.

### 4.1 Menstrual cycle characteristics

The average age of menarche in this study (13.21 years) aligns with findings from several studies, which consistently report delayed menarche in female athletes compared to non-athletes (9, 10, 19, 20).

Menstrual cycle regularity was observed in approximately 80% of the participants, with a mean length of 27.69 ± 3.78 days. This finding is similar to those published in other studies made over athletic (16, 19), and general population (21).

In 2022, Vannuccini et al. noted that approximately 30% of the general female population experienced heavy menstrual bleeding at some point during their reproductive life (22). In our study, this percentage was significantly higher (55.70%). This figure also exceeds the percentage obtained in other studies over sports population where the results moved from 30 to 40% (10, 19, 23). This difference may be influenced by the fact that it is a subjective data, in which the players transmit what they perceive without having a standard that serves as a measure.

The main symptom reported during the period was dysmenorrhea. This is another highly variable figure in the existing literature. Ju et al. determined that 20–90% of women present dysmenorrhea (24), while for Wotjys et al. this range goes from 16 to 91% (12). The ranges of dysmenorrhea are so wide that it is not possible to get a clear picture of the problem. The results in this study are similar to those described by Armour et al. (19) and Findlay et al. (10) in elite sports populations with 82.3% and 80% of dysmenorrhea among their players.

Dysmenorrhea data of the Spanish players coincides with data found in the literature. Armour et al. also described pain on the first day as being more common and less common for pain to persist throughout menstruation (19).

After dysmenorrhea, back pain was the second most common symptom (39.47%). This aligns with most studies on both general and sports populations (15), except for Findlay et al., who described that only 6.67% of female rugby players reported back pain (10). The authors suggest this discrepancy could be attributed to the small sample size or the likelihood that athletes in contact sports often experience back pain unrelated to menstruation. However, since basketball is a contact sport too, our study challenges the latter idea.

Back pain is followed in frequency by changes in appetite (31.14%) and fatigue (29.82%). Fatigue has also been observed in the studies by Findlay et al. (10) and Armour et al. (19).

Nearly half of the female basketball players surveyed reported taking pain medication during menstruation, data similar to the findings of Armour et al. (19) and Findlay et al. (10) in their studies.

PMS is also prevalent in our study (77.63%). These data are similar to those found in the studies by Findlay et al. (10) and Bruinvels et al. (23) and the ones observed by Amour's group (19). The most frequent symptoms among the participants in our study were breast tenderness and swelling, mood swings, and general swelling; similarly to what was observed by Armour et al. (19).

If, like other authors, we consider the symptoms of menstruation and PMS together, the most repeated symptoms apart from dysmenorrhea are variations in appetite, breast tenderness, fatigue, mood swings, back pain, and bloating, coincident with results in non-professional sports population (23) and elite sports population (10).

A percentage of 22.81% of the respondents, reported having amenorrhea at some point in their lives. This percentage is quite high when compared to the studies by Findlay et al., in which no female players reported having amenorrhea (10), or Martin et al., in which less than 1% of female players reported having it (25). This difference could be due to the small study population used by Findlay et al. (10) and due to the fact that Martin et al. (25) may underestimated amenorrhea because it was not a question that appeared in the questionnaire, and the only 3 players who reported amenorrhea stated it as an extra piece of information. It is difficult to find relevant information about amenorrhea in the articles consulted since amenorrhea is often an exclusion criterion in studies about MC. In this study, the cause of amenorrhea is unknown for 42.59% of the players, while 29.63% relate it to stress, and 11.11% to sudden weight loss. This stratification of causes is inverted if we look at the causes by leagues, with stress being the main cause in LF1 players and even more accused in LF1E players, the most professional players with the highest sporting demands.

A percentage of 3.51% of the players in our study, reported having been pregnant at some point in their lives. This figure is notably lower than the national average for women between 25 and 29 years old (20.8% have given birth) as reported by the National Institute of Statistics in 2018 (26).

The figure of our study is closer to the 2% of professional footballers up to 33 years who reported being mothers in the 2017 Global Employment Report of the International Federation of Professional Footballers (27), reflecting the fact that motherhood is delayed in general in the world of professional sports and not only in basketball. This may be attributed to the limited implementation of work-life balance policies in professional sports. However, in recent years this situation has begun to change: the new International Federation of Association Football (FIFA) policies have included maternity policies since 2021 (28); also, the professional football club *Associazione Calcio Milan* now includes measures favoring safety and work-life balance during pregnancy and early childhood (29).

Regarding less frequent gynecological disorders, the most frequent alteration was polycystic ovarian syndrome (PCOS) being much more frequent among LF1E players, the highest-level players in the study. However, given the small number of the PCOS cases, it is not possible to determine whether there is any relationship between PCOS and sports performance. There are numerous publications describing physical activity and sport as beneficial in combating PCOS symptoms (31–33), but none linking PCOS to sports performance, therefore, more studies are needed to determinate if there is any relation between this or not. The data from the Findlay et al. study (10) was slightly higher (6,67%) than the obtained in the present study (2.63%). Finally, according to the World Health Organization (30), in the general population, between 8% and 13% of women of reproductive age experience PCOS, which is triple the figure presented in this study. Our lower incidence may be due to an underestimation as a result of the low use of women's reproductive health services among the players. In agreement with our data, Findlay et al. (10) reported that, before going to the gynecologist, the female players surveyed first consult their general practitioner or sports doctor for advice on problems related to the MC.

### 4.2 Hormonal contraceptive use

We found that the use of HCs is not very widespread among the LF1 and LF2 basketball players surveyed. Less than a quarter of the surveyed players reported using them and this percentage decreases even more among the LF1E players. Our data differs from other sports populations as 47.1% and 49.5% of the elite athlete surveyed by Larsen et al. (16) and Martin et al. (15), were using oral contraceptive pills. This could be due to an overestimation of the use of HCs in those study populations, as their numbers are higher than others found in the literature. However, our data is more consistent with the27% use described in Findlay et al. study (10), and with the data found in the general population, where the use of oral contraceptive pills is around 30% (14, 34). Our data is still slightly lower than these data, so we think that further studies may be needed to analyze whether there is currently a trend among young people to reduce the use of hormonal contraceptives or is just a particularity of this group.

Our results also align with the existing literature on the HCs of choice. The oral pill was the most frequent used HCs in our study (74.07%) which is close to the 78.4% of sportswomen in Martin's study (15) and 75.3% in Larsen's study (16) who also prefer the oral pill. However, there is a change in this trend among the group of LF1E players, where the majority chose non-oral HCs. This change in the trend is not significant given that the number of players using HC within the LF1E group is very small, so more studies could be done to establish if the type of HC is related to the level of professionalism in sports.

Between basketball players who took HCs and those who did not, no differences were found in PMS, but a decrease in dysmenorrhea was observed: only 28.30% of players taking HCs reported suffering from it, compared with 48.00% of players not taking HCs. This is consistent with the scientific data that support the use of HC as a treatment for dysmenorrhea (35, 36). There are also differences in the regularity of the cycle, with 83.02% of the players taking HCs having irregular MC, compared to 77.14% of those not taking HCs. This data does not align with what has been observed in the literature, since one of the described effects of HC use is cycle regularity (36). This may be because the players may have responded based on the regularity of their cycle when not taking medication (HCs). It would be interesting in future studies to clearly define the interpretation of this question.

Data were self-reported, which may introduce response bias and affect accuracy; however, rigorous data-collection procedures and the large study population likely mitigated this risk.

Furthermore, to our knowledge, no prior studies have specially characterized the MC in female elite basketball players.

As this study was conducted on a population of elite female basketball players in Spain; it would be interesting to replicate the study in other athletic population to examine whether similar results are observed.

## 5 Conclusions

Over three-quarters (86.84% and 77.63%) of the female athletes surveyed reported dysmenorrhea and premenstrual syndrome, respectively; and over half of the players declared having heavy menstrual bleeding during their periods.

One-quarter of the players included in the study referred having amenorrhea at some point during their menstrual life.

Less than 5% of the players had ever been pregnant.

One-quarter of the participants reported using hormonal contraceptives. The most commonly used (23.25%) was the oral pill.

## Data Availability

The raw data supporting the conclusions of this article will be made available by the authors, without undue reservation.
