# Supplementary material for: Characteristics of the menstrual cycle and hormonal contraceptive use in elite Spanish basketball players
Source: Front Sports Act Living. 2025 Oct 29;7:1642415. doi: 10.3389/fspor.2025.1642415 (PMC12605390; doi:10.3389/fspor.2025.1642415)
Supplement: Supplementary file 1 [file Datasheet1.pdf]

Survey on performance and menstrual cycle in women's basketball

Thank you very much for collaborating and helping us learn more about the influence of the menstrual cycle on the performance of basketball players. You just have to answer some questions to get to know your menstrual cycle better.

* Indicates that the question is mandatory

1. First Name *

____

Example: CADI7

For confidentiality put the coding of your equipment as it appears next, followed by your jersey number

Cadi La Seu: CADI;
Campus Promete: CAMP;
Ciudad de los adelantados: CIUD;
Durán Maquinaria Ensino: ENSI;
Embutidos Pajariel Bembibre PDM: BEMB;
IDK Guipuzcoa: IDK
Lointek Gernika Bizkaia: LOIN;
Mann Filtar Casablanca: MANF;
Nissan Al-Qázeres Extremadura: ALQA;
Perfumerías Avenida: PERF;
Quesos el Pastor: QUES;
RPK Araski: RPK;
Spar Citylift Girona: SPAR;
Valencia B. C.: VALE

2. Mail

We will only use this email to send you another form that will help us register and keep track of the day you get your period every month

____

3. Position

Select all that apply

- ☐ Point guard
- ☐ Shooting guard
- ☐ Small forward
- ☐ Power forward
- ☐ Center

4. Are you right or left-handed?

Select all that apply

- [ ] Right-handed
- [ ] Left-handed
- [ ] Ambidextrous

5. Date of birth

____

Example: January 7, 2019

Menstrual cycle

Please, answer the questions in reference to last year

6. Age of first period

____

7. Date of your period in September *

____

Example: January 7, 2019

8. Is your period regular?

Mark only one option

- [ ] Yes
- [ ] No

9. Your menstrual cycle lasts (days between one period and the next one)

Mark only one option

- [ ] 21 days
- [ ] 28 days
- [ ] 30 days
- [ ] 32 days
- [ ] 40 days
- [ ] Other: ______________________________

10. Are your periods painful?

Mark only one option

- ☐ Never
- ☐ More than 6 cycles ago they were, but not anymore
- ☐ Sometimes, just the first day
- ☐ Sometimes just the first and second days
- ☐ Always, the first day
- ☐ Always, the first and second days
- ☐ During the whole period
- ☐ Other: ______________________________

11. Do you have any other symptoms during your period?

Select all that apply

- ☐ No, anyone
- ☐ Nausea
- ☐ Low tension
- ☐ Gastrointestinal symptoms (diarrhea/constipation)
- ☐ Back pain
- ☐ Thighs pain and weakness
- ☐ Abdominal cramps
- ☐ Sweating
- ☐ Headache
- ☐ Fatigue
- ☐ Appetite changes
- ☐ Other: ______________________________

12. Do you have PMS (premenstrual syndrome) the five days before the period?

Select all that apply

- ☐ No, I have no symptoms
- ☐ Nausea
- ☐ Breast swelling and tenderness
- ☐ Bloating
- ☐ Mood swings (bad temper, depression, apathy...)
- ☐ Fatigue
- ☐ Appetite changes
- ☐ Other: ______________________________

13. Have you ever skipped 2 or more periods?

Mark only one option

- [ ] Ever
- [ ] Never

14. If yes, do you know why?

Select all that apply

- [ ] No, I don´t know why
- [ ] Sudden weight loss
- [ ] Physical or mental stress
- [ ] Depression
- [ ] Other condition

15. If yes, how was it solved?

____

16. My menstrual flow is very heavy

Mark only one option

- [ ] Yes
- [ ] No
- [ ] Sometimes

17. My periods are 7 or more days long

Mark only one option

- [ ] Yes
- [ ] No
- [ ] Sometimes

18. Do you have any other gynecological or endocrine disorders?

Mark only one option

- [ ] Yes
- [ ] No

19. If yes, which one?

___

20. How many times have you been pregnant? (both born and abortions)

___

21. Do you take contraceptives?

Mark only one option

- [ ] Yes
- [ ] No

22. If yes, what kind and since when?

___

23. Do you usually take any medication?

Mark only one option

- [ ] Yes
- [ ] No

24. If yes, what kind and since when?

___

25. Do you take any medication during your period?

Mark only one option

- [ ] Yes
- [ ] No

26. If yes, what kind and since when?

___

☐

27. Do you do gynecological checkups?

☐ Annual

☐ Every two years

☐ When I have a problem

☐ Never

Thank you very much for your cooperation!!

Thank you for dedicating part of your valuable time, this information is very useful for us to continue improving the performance of women athletes
