# Supplementary material for: Characteristics of the menstrual cycle and hormonal contraceptive use in elite Spanish basketball players
Source: Front Sports Act Living. 2025 Oct 29;7:1642415. doi: 10.3389/fspor.2025.1642415 (PMC12605390; doi:10.3389/fspor.2025.1642415)
Supplement: Supplementary file 2 [file Datasheet2.pdf]

Encuesta sobre rendimiento y ciclo menstrual en el baloncesto femenino

Muchas gracias por colaborar y ayudarnos a conocer más la influencia del ciclo menstrual en el rendimiento de las jugadoras de baloncesto. Sólo tienes que contester a unas preguntas para poder conocer major tu ciclo menstrual.

* Indica que la pregunta es obligatoria

1. Nombre*

____

Ejemplo: CADI7

Por confidencialidad, pon la codificación de tu equipo tal como aparece a continuación, seguida de tu número de camiseta de juego

Cadi La Seu: CADI;
Campus Promete: CAMP;
Ciudad de los adelantados: CIUD;
Durán Maquinaria Ensino: ENSI;
Embutidos Pajariel Bembibre PDM: BEMB;
IDK Guipuzcoa: IDK
Lointek Gernika Bizkaia: LOIN;
Mann Filtar Casablanca: MANF;
Nissan Al-Qázeres Extremadura: ALQA;
Perfumerías Avenida: PERF;
Quesos el Pastor: QUES;
RPK Araski: RPK;
Spar Citylift Girona: SPAR;
Valencia B. C.: VALE

2. Mail

Este email solo se usará para mandarte otro formulario que nos sirva para poder registrar y llevar control del día que te baja la regla cada mes

____

3. Puesto de juego actualmente

Selecciona todas las que correspondan

- [ ] 1
- [ ] 2
- [ ] 3
- [ ] 4
- [ ] 5

4. ¿Eres diestra o zurda?
Selecciona todas las que correspondan

- [ ] Diestra
- [ ] Zurda
- [ ] Ambidiestra

5. Fecha de nacimiento

___

Ejemplo: Enero 7, 2019

Ciclo menstrual

Por favor, contesta a las preguntas en referencia al último año

6. Edad de la primera regla

___

7. Fecha de regla en septiembre *

___

Ejemplo: Enero 7, 2019

8. ¿Es regular?
Marcar solo una opción

- [ ] Si
- [ ] No

9. La duración de tu ciclo menstrual es (días entre una regla y otra)
Marcar solo una opción

- [ ] 21 días
- [ ] 28 días
- [ ] 30 días
- [ ] 32 días
- [ ] 40 días
- [ ] Otra: ___

10. ¿Tus menstruaciones son dolorosas?

Marcar solo una opción

- [ ] Nunca
- [ ] Hace más de 6 ciclos lo eran, pero ya no
- [ ] Alguna vez, pero solo el primer día
- [ ] Alguna vez, pero solo el primer y el segundo día
- [ ] Siempre el primer día
- [ ] Siempre el primer y segundo día
- [ ] Durante toda la menstruación
- [ ] Otra: ____

11. ¿Tienes algún otro síntoma durante las menstruaciones?

Selecciona todas las que correspondan

- [ ] No, ningún síntoma
- [ ] Náuseas
- [ ] Tensión arterial baka (hipotensión)
- [ ] Trastornos intestinales (diarreas, malestar…)
- [ ] Dolor lumbar
- [ ] Dolos y debilidad en los muslos
- [ ] Dolor abdominal
- [ ] Sudoraciones
- [ ] Dolor de cabeza
- [ ] Fatiga
- [ ] Cambios en el apetito
- [ ] Otra: ____

12. Los cinco días previos a la regla, ¿tienes algún síntoma premenstrual?

Selecciona todas las que correspondan

- [ ] No, ningún síntoma
- [ ] Náuseas
- [ ] Dolor e inflamación de las mamas
- [ ] Retensión de líquidos, aumento de peso o sentirse hinchada
- [ ] Alteraciones del estado emocional: mal humor, depresión, apathie...
- [ ] Fatiga
- [ ] Cambios en el apetito
- [ ] Otra: ____

13. ¿Has dejado de tener la regla durante 2 o más ciclos?

Marcar solo una opción

- ☐ Alguna vez
- ☐ Nunca

14. En caso afirmativo, ¿sabes la causa?

Selecciona todas las que correspondan

- ☐ No, desconozco la causa
- ☐ Pérdida de peso brusca
- ☐ Estrés físico o mental
- ☐ Depresión
- ☐ Otra enfermedad

15. En caso afirmativo, ¿cómo se solucionó?

16. Mis menstruaciones son demasiado abundantes en cantidad:

Marcar solo una opción

- ☐ Si
- ☐ No
- ☐ A veces

17. Mis menstruaciones son demasiado largas (7 o más de 7 días)

Marcar solo una opción

- ☐ Si
- ☐ No
- ☐ A veces

18. ¿Alguna otra alteración ginecológica o endocrina?

Marcar solo una opción

- ☐ Si
- ☐ No

19. En caso afirmativo, ¿cuál?

____

20. Señala el número de veces que te has quedado embarazada, hayas tenido un hijo o haya finalizado en aborto

____

21. ¿Tomas anticonceptivos?

Marcar solo una opción

☐ Si

☐ No

22. En caso afirmativo, ¿qué tipo y desde cuándo?

____

23. ¿Tomas alguna otra medicación actualmente?

Marcar solo una opción

☐ Si

☐ No

24. En caso afirmativo, ¿qué tipo y desde cuándo?

____

25. ¿Tomas alguna medicación durante la regla?

Marcar solo una opción

☐ Si

☐ No

26. En caso afirmativo, ¿qué tipo y desde cuándo?

____

☐

27. ¿Haces revisions ginecológicas?

- ☐ Anual
- ☐ Cada dos años
- ☐ Cuando tengo algún problema
- ☐ Nunca

¡Muchas gracias por tu colaboración!

Gracias por dedicarnos parte de tu valioso tiempo, esta información nos es muy útil para seguir mejorando el rendimiento en la mujer deportista.
