# Supplementary material for: Characteristics of the menstrual cycle and hormonal contraceptive use in elite Spanish basketball players
Source: Front Sports Act Living. 2025 Oct 29;7:1642415. doi: 10.3389/fspor.2025.1642415 (PMC12605390; doi:10.3389/fspor.2025.1642415)
Supplement: Supplementary file 3 [file Datasheet3.pdf]

Enquête sur la performance et le cycle menstruel dans le basketball féminin

Merci beaucoup pour votre collaboration et de nous avoir aidés à en apprendre davantage sur l'influence du cycle menstruel sur la performance des joueurs de basketball. Ces questions sont importantes pour mieux comprendre les caractéristiques du cycle menstrual.

* Indique que la question est obligatoire

1. Nom*

____

Exemple: CADI7

Pour des raisons de confidentialité, indiquez le codage de votre équipement tel qu'il apparaît à côté, suivi de votre numéro de maillot

Cadi La Seu: CADI;
Campus Promete: CAMP;
Ciudad de los adelantados: CIUD;
Durán Maquinaria Ensino: ENSI;
Embutidos Pajariel Bembibre PDM: BEMB;
IDK Guipuzcoa: IDK
Lointek Gernika Bizkaia: LOIN;
Mann Filtar Casablanca: MANF;
Nissan Al-Qázeres Extremadura: ALQA;
Perfumerías Avenida: PERF;
Quesos el Pastor: QUES;
RPK Araski: RPK;
Spar Citylift Girona: SPAR;
Valencia B. C.: VALE

2. Mail

Ce mail ne sera utilisé que pour vous envoyer un autre formulaire qui nous aidera à vous inscrire et à garder votre cycle menstruel

____

3. Position du jeu actuellement

Sélectionnez tout ce qui s´applique

- [ ] 1
- [ ] 2
- [ ] 3
- [ ] 4
- [ ] 5

4. Vous êtes droitier or gaucher?

Sélectionnez tout ce qui s´applique

- [ ] Droitière
- [ ] Gauchère
- [ ] Ambidextre

5. Date de naissance

____

Exemple: Janvier 7, 2019

Cycle menstruel

S’il vous plait, répondez aux questions en reference à l’année dernière

6. Âge de la première règle

____

7. Date de votre règle en septembre *

____

Exemple: Janvier 7, 2019

8. Ton cycle menstrual est régulier?

Choisissez une seule option

- [ ] Oui
- [ ] Non

9. La durée de votre cycle menstrual est de (jours entre une règle et la suivante)

Choisissez une seule option

- [ ] 21 jours
- [ ] 28 jours
- [ ] 30 jours
- [ ] 32 jours
- [ ] 40 jours
- [ ] Autre: ______________________________

10. Vos règles sont-elles douloureuses?

Choisissez une seule option

- [ ] Jamais
- [ ] Il y a plus de six cycles qu'elles l'etaient, mais plus maintenant
- [ ] Quelque fois, mais seulement le premier jour
- [ ] Quelque fois, mais seulement le premier et le deuxième jour
- [ ] Toujours, le premier jour
- [ ] Toujours, le premier et le deuxième jour
- [ ] Tout a long des menstruation
- [ ] Autre: ______________________________

11. Avez-vous d'autres symptoms pendant la menstruation?

Sélectionnez tout ce qui s´applique

- [ ] No, pas de symptômes
- [ ] Nausées
- [ ] Basse pression artérielle (hypotension)
- [ ] Troubles intestinaux (diarrhée, malaise…)
- [ ] Douleur lombaire
- [ ] Douleur et faiblesse dans les cuisses
- [ ] Douleur abdominale
- [ ] Sudation
- [ ] Mal de tète
- [ ] Fatigue
- [ ] Changements d'appétit
- [ ] Autre: ______________________________

12. Les cinq jours precedent vos règles, avez-vous des sympômes prémenstruels?

Sélectionnez tout ce qui s´applique

- [ ] Non, pas de symptômes
- [ ] Nausées
- [ ] Douleur et gonflement des seins
- [ ] Rétention hydrique, prise de poids ou sensation de ballonnement
- [ ] Altérations de l'état émotionnel (mauvaise humeur, dépression, apathie...)
- [ ] Fatigue
- [ ] Changements d'appétit
- [ ] Autre: ______________________________

13. Avez-vous cessé d’avoir vos règles pendant 2 cycles ou plus?

Choisissez une seule option.

- [ ] Parfois
- [ ] Jamais

14. Si oui, connaisez-vous la cause?

Sélectionnez tout ce qui s´applique

- [ ] Non, je ne connais pas la cause
- [ ] Perte de poids brusque
- [ ] Stress physique ou mental
- [ ] Dépression
- [ ] Une autre maladie

15. Si oui, comment a-t-il été résolu?

____

____

____

____

____

16. Mes règles sont trop abondantes en quiantité:

Choisissez une seule option

- [ ] Oui
- [ ] Non
- [ ] Parfois

17. Mes règles sont trop longues (7 ou plus de 7 jours)

Choisissez une seule option

- [ ] Oui
- [ ] Non
- [ ] Parfois

18. Avez-vous dáutres troubles gynécologiques ou endocriniens?

Choisissez une seule option

- [ ] Oui
- [ ] Non

19. Si oui, lequel?

____

20. Combien de fois avez-vous été enceinte ? (naissances et avortements)

____

21. Prenez-vous des contraceptifs?

Choisissez une seule option

- [ ] Oui
- [ ] Non

22. Si oui, de quell type et depuis quand?

____

23. Prenez-vous habituellement un autre medicament?

Choisissez une seule option

- [ ] Oui
- [ ] Non

24. Si oui, de quell type et depuis quand

____

25. Prenez-vous des medicaments pendant vos règles?

Choisissez une seule option

- [ ] Oui
- [ ] Non

26. Si oui, de quell type et depuis quand?

____

☐

27. Faites-vous des examens gynécologiques?

☐ Annuellement

☐ Tous les deux ans

☐ Quand j’ai un problème

☐ Jamais

Merci beaucoup pour votre collaboration!!
Merci de consacrer un peu de votre précieux temps, ces informations nous sont très utiles pour continuer à améliorer les performances des athlètes féminines.
